# Membrane-Binding Mechanism of *Clostridium perfringens* Alpha-Toxin

**DOI:** 10.3390/toxins7124880

**Published:** 2015-12-03

**Authors:** Masataka Oda, Yutaka Terao, Jun Sakurai, Masahiro Nagahama

**Affiliations:** 1 Division of Microbiology and Infectious Diseases, Niigata University Graduate School of Medical and Dental Sciences, 2-5274, Gakkocho-dori, Chuo-ku, Niigata 951-8514, Japan; terao@dent.niigata-u.ac.jp; 2 Department of Microbiology, Faculty of Pharmaceutical Sciences, Tokushima Bunri University, Yamashiro-cho, Tokushima 770-8514, Japan; sakuraijun7400@gmail.com (J.S.); nagahama@ph.bunri-u.ac.jp (M.N.)

**Keywords:** *Clostridium perfringens*, alpha-toxin, *C*-domain, central loop domain, phospholipid, ganglioside GM1a, TrkA, endocytosis, vaccine

## Abstract

*Clostridium perfringens* alpha-toxin is a key mediator of gas gangrene, which is a life-threatening infection that manifests as fever, pain, edema, myonecrosis, and gas production. Alpha-toxin possesses phospholipase C and sphingomyelinase activities. The toxin is composed of an *N*-terminal domain (1–250 aa, *N*-domain), which is the catalytic site, and a *C*-terminal domain (251–370 aa, *C*-domain), which is the membrane-binding site. Immunization of mice with the *C*-domain of alpha-toxin prevents the gas gangrene caused by *C. perfringens*, whereas immunization with the *N*-domain has no effect. The central loop domain (55–93 aa), especially H….SW^84^Y^85^….G, plays an important role in the interaction with ganglioside GM1a. The toxin binds to lipid rafts in the presence of a GM1a/TrkA complex, and metabolites from phosphatidylcholine to diacylglycerol through the enzymatic activity of alpha-toxin itself. These membrane dynamics leads to the activation of endogenous PLCγ-1 via TrkA. In addition, treatment with alpha-toxin leads to the formation of diacylglycerol at membrane rafts in ganglioside-deficient DonQ cells; this in turn triggers endocytosis and cell death. This article summarizes the current the membrane-binding mechanism of alpha-toxin in detail.

## 1. Introduction

*Clostridium perfringens* alpha-toxin is an important agent in gas gangrene [1,2], and causes hemolysis, platelet aggregation, contraction of blood vessels, superoxide generation, cytokine storm, and ultimately death [3,4,5,6,7,8,9]. The research about structure-function relationship of alpha-toxin has been reported much until now [4,9,10,11,12]. Comparative analysis of the putative amino acid sequences encoded by the genes for alpha-toxin and *Bacillus cereus* PLC indicates that alpha-toxin belongs to a family of bacterial zinc-metallo phospholipase C enzymes [9]. Alignment of the amino acid sequences of these enzymes reveals two groups: the single-domain proteins and the two-domain proteins. The single-domain proteins (*Listeria monocytogenes* PLC and *Bacillus cereus* PLC) consist of approximately 250 amino acids [13]. The two-domain proteins (*Clostridium novyi* gamma-toxin, *Clostridium bifermentans* PLC, and *C. perfringens* alpha-toxin) are typically composed of approximately 370 amino acids.

Based on crystallographic data and a site-directed mutagenesis analysis, the relationship between the alpha-toxin amino acid residues, co-ordination of zinc ions, and biological activity has been revealed [4]. One zinc ion is tightly coordinated with Trp-1, His-11, and Asp-130, a second is coordinated with His-148 and Glu-152, and a divalent cation is associated with His-68, -126, -136, and Asp-130 [4]. These results indicate that the catalytic site of the toxin is located in the *N*-domain. While the presence of the *C*-domain (approximately 120 aa) is correlated with a hemolytic activity of the enzymatic domain, it is not required for enzyme activity.

Drawing on studies of alpha-toxin’s biological properties, structure-function, and mode of action, this review summarizes current findings, discusses the membrane-binding mechanism of alpha-toxin.

## 2. Role of *C*-Terminal Domain in the Binding of Alpha-Toxin to Membrane

**Figure 1:**
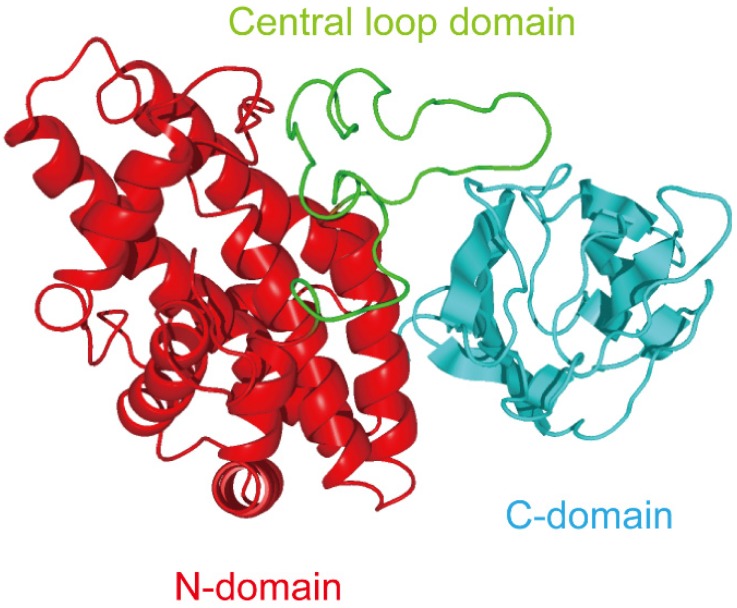
Three domains of *Clostridium perfringens* alpha-toxin.

Three-demensional analysis of alpha-toxin revealed that the structure is divided into two domains [10]: the *N*-domain, which consists of nine tightly packed α-helices, and the *C*-domain, which consist of an eight-stranded antiparallel β-sandwich motif (Figure 1). The *C*-domain is known as the PLAT (Polycystin-1, Lipoxygenase, Alpha-toxin) domain, is a membrane binding domain [14]. This structure forms a beta-sandwich composed of two sheets of four strand each. Naylor *et al.* reported that Asp-269 and -336 specifically interact with calcium ions [15]. Furthermore, mutational analysis revealed that Tyr-275, -307, -331, and Phe-334 residues are critical for binding of the toxin [16,17]. Experiments using acrylodan-labeled alpha-toxin showed that Ser263 and Ser365 of the *C*-domain are inserted into phospholipid bilayer membranes [18]. It seems that the *C*-domain plays a role in binding to membranes. The binding of toxin to liposomes, and the subsequent toxin-dependent hydrolysis of phosphatidylcholine in liposomes, is also related to the phase transition temperature of the PC [18,19]. The membrane-damaging action of alpha-toxin is dependent on membrane fluidity, and invasion of the *C*-domain into the bilayer membrane may play an essential role in its action.

## 3. Exploitation of *C*-Terminal Domain of Alpha-Toxin

*C. perfringens* type A toxemia in mammalian hosts proceeds through wounds or via the orogastric route, depending on the release of toxins. Therefore, the protection against type A toxemia would ideally be achieved using a vaccine molecule capable of generating both systemic and mucosal immunity, since this would neutralize the soft tissue and intestinal damage induced by alpha-toxin. The *N*-domain (CP1-249) and *C*-domain (CP247-370) of alpha-toxin were produced using genetic engineering of *E. coli* as candidate vaccines [20]. Mice immunized with CP1-249 died following an alpha-toxin challenge. However, mice immunized with CP247-370 were protected against challenge with either toxin or *C. perfringens* type A in an experimental gas gangrene model [20,21]. Stevens *et al.* [22] reported that active immunization with CP247-370 was effective in a mouse model of gas gangrene. Immunization with CP247-370 restricted the spread of infection and prevented ischemia of the feet. Histopathological findings revealed limited muscle necrosis, reduced microvascular thrombosis, and enhanced granulocytic influx in CP247-370-immunized mice [22]. Recently, Shreya *et al.* reported that mice immunized with a bivalent chimeric protein (composed of the *C*-domains of alpha-toxin and enterotoxin from *C. perfringens*) resulted in substantial protection against *C. perfringens* type A toxemia [23]. Therefore, the *C*-domain of the toxin may be the principal immunogen of an alpha-toxin vaccine. To determine which region of the alpha-toxin *C*-domain was the strongest immunogen, we investigated the efficacy of active immunization with various fragments. We determined that either of two *C*-terminal alpha-toxin fragments (CP251-370 or CP281-370) could protect against clostridial myonecrosis and diseases in which the *C. perfringens* alpha-toxin is a major virulence factor [24,25].

## 4. Relationship between Alpha-Toxin and Gangliosides

**Figure 2:**
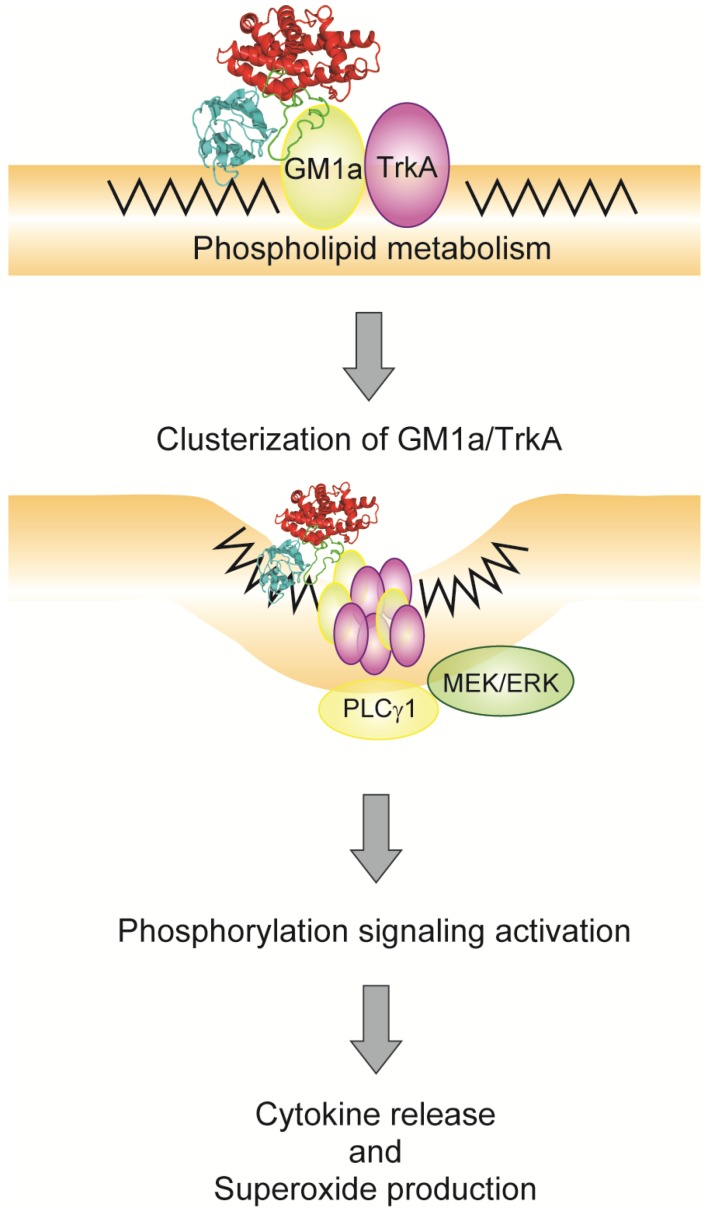
Model of alpha-toxin-induced signal transduction via GM1a/TrkA.

Alpha-toxin has two flexible loops [10,26]. These two loops are on either side of the active site cleft, on the face of the toxin molecule that is believed to interact with the membrane surface [10]. The first of these loops (residues 55–93; central loop domain, Figure 1) lies between the active site cleft and the cleft between the *N*- and *C*-terminal domain [26]. The second loop (residues 132–149) contains residues involved in the binding of the third zinc ion [26]. Clark *et al.* [27] previously suggested that the 72–93-residue loop in alpha-toxin participates in membrane binding. Previous experiments with tetanus neurotoxin (TeNT) and botulinum neurotoxin (BoNT) found that the lactose- and ganglioside-GT1b-binding site is characterized by the presence of the peptide motif H…SXWY…G (approximately 30 aa), with the tryptophan and tyrosine residues being especially important [28]. A similar motif, such as H…SWY…G (residues 68–93; 26 aa), is conserved in alpha-toxin [29]. We have produced single-point mutants, which are substitution of six amino acid residues (Asp-81, Asn-82, Ser-83, Trp-84, Tyr-85, Tyr-88) to alanine, to further define the molecular interaction between alpha-toxin and GM1a [29]. Substitution of Trp-84 and Tyr-85 residues coordinated in positions comparable to the ganglioside binding pockets of TeNT and BoNTs dramatically affected the binding to GM1a-liposome [29]. In addition, we showed that the W84A and Y85A variants were significantly reduced in their ability to activate TrkA [29]. GM1a associates with TrkA on cell membranes, and activates TrkA and ERK1/2 [30,31,32]. Cholera toxin, which is a high-affinity ligand for GM1, activated TrkA in PC12 cells [31]. Ichikawa *et al.* [33] reported that GM1 clustering promotes the enrichment of TrkA in the lipid raft and activation of downstream signal transduction pathways. We also found that alpha-toxin induced diacylglycerol formation at the plasma membrane [34]. The ensuing flip-flop motion of diacylglycerol influences membrane dynamics, thereby promoting GM1a clustering and activation of endogenous phospholipase C-γ1 via TrkA [34,35]. These results suggest that the binding of alpha-toxin to GM1a plays an important role in the clustering and activation of TrkA (Figure 2).

We analyzed the mode of action of binding between central loop domain, especially Trp-84 and Tyr-85, of alpha-toxin (PDB; 1CA1) and carbohydrate moiety of GM1a by *in silico* docking simulation analyses of this interaction at the tertiary structure level using the MOE software. The results of our current *in silico* docking analyses show that Trp-84 interacts with the sialic acid of GM1a through a hydrophobic ring-stacking mechanism and a hydrogen bond, and Tyr-85 interacts with the galactosamine of GM1a through a hydrogen bond [29]. The structures of BoNT and ganglioside complexes show that the indole ring of the tryptophan residue in the loop motif is crucial for the structural integrity of the sialic acid-binding site [36,37,38]. Thus, our results support the hypothesis that Trp-84 of alpha-toxin plays an important role in the interaction with the sialic acid of GM1a. The *C*-domain (PLAT domain) of alpha-toxin contains a phospholipid-binding site [10,18,39]; our results suggest that the ganglioside-binding site in the loop domain plays an important role in the tethering of alpha-toxin to membrane.

## 5. Endocytosis of Alpha-Toxin in Ganglioside Deficient Cells

Monturiol-Gross *et al.* reported that the endocytosis of alpha-toxin is required for the activation of the MEK/ERK pathway and the subsequent cytotoxicity [40]. Cholesterol is a key component of caveolae, and MβCD, nystatin, and filipin have been used in pharmacological approaches for study these lipid domains [41,42]. Depleting cholesterol from the plasma membrane significantly increased survival of DonQ cells, which are ganglioside deficient cells, challenged with alpha-toxin [40]. We reported that alpha-toxin binds almost exclusively to detergent-resistant membranes [43]. Several treatments that affect glycosylphosphatidyl-inositol anchored proteins (GPI-AP), which are associated with cholesterol-enriched domains [44], also protect cells from the cytotoxic effect of alpha-toxin. However, it is still not clear whether alpha-toxin binds to a GPI-AP as a receptor or coreceptor or GPI-AP is involved in alpha-toxin internalization. The prosurvival effect of cholesterol depletion may be because the process impairs alpha-toxin endocytosis: perhaps an intact cholesterol-rich domain is required for binding of the toxin to cell surface receptors [40].

The labeled toxin can be seen as characteristic vesicles of some endocytic pathways, such as clathrin, caveolae and Rho A-dependent endocytosis [40,45]. Furthermore, dynamin (Dyn)-dominant negative cells have reduced numbers of intracellular vesicles containing alpha-toxin [40]. Dyn-dependent endocytic pathways include clathrin, caveolae, flotillin, and Rho A-dependent endocytosis [45,46]. Labeled alpha-toxin does not colocalize with clathrin on DonQ cells, but colocalizes with caveolin-1 at the plasma membrane and in intracellular vesicles, suggesting a role for a caveolar-type endocytosis during toxin internalization. This coincides with recent studies demonstrating that alpha-toxin binds to GM1a as a receptor [29], because GM1 is endocytosed via caveolae [47]. It is possible that the same endocytic cargo may be internalized by different mechanisms in different cell types, or may change the pathways in a single cell type under different conditions [41].

**Figure 3:**
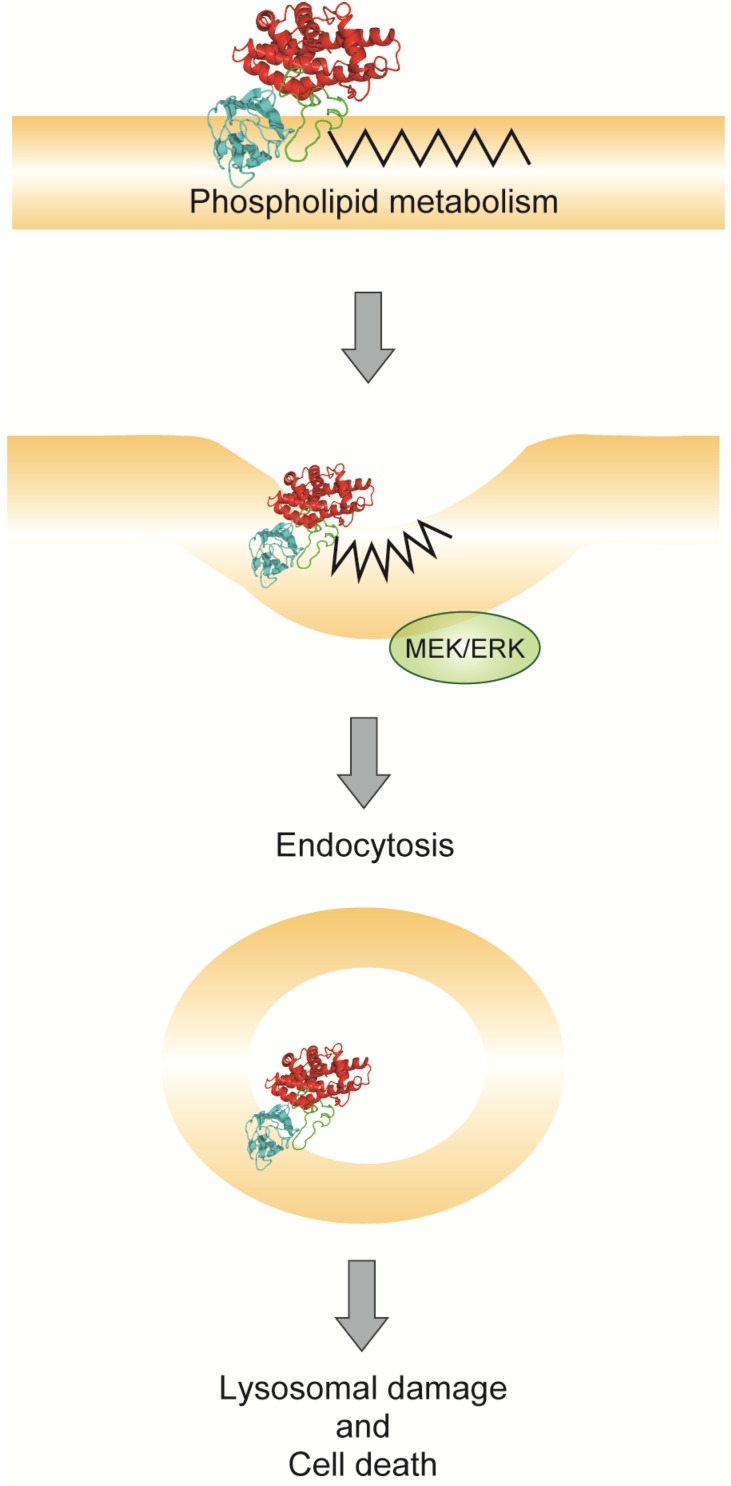
Model of alpha-toxin-induced endocytosis.

The enzymatic activity of alpha-toxin leads to the formation of diacylglycerol and ceramide on the plasma membrane [4,35]. Its enzymatic activity induces negative curvature, which promotes the endocytic uptake of the bilayer-like diacylglycerol [48]. The transient formation of diacylglycerol and ceramide in non-bilayer membrane intermediates promotes membrane fusion [49,50]. We have previously shown that alpha-toxin variants with single point substitutions, such as H148G, have dramatically reduced cytotoxic activity [51,52]. Therefore, we suggested that diacylglycerol and ceramide generation in the ganglioside deficient cells treated with alpha-toxin leads to endocytosis and cell death (Figure 3).

## 6. Conclusions

The central loop domain and *C*-domain of alpha-toxin play important roles in the binding to ganglioside GM1a and phospholipids, respectively. We suggested that binding of alpha-toxin to GM1a is the tethering step, while binding to phospholipids is the colonizing step. Alpha-toxin-dependent stimulation of TrkA signal transduction depends on gangliosides, but endocytosis of the toxin does not. The biological consequences of alpha-toxin binding vary according to the cell type or status. Currently, our understanding of the effect of alpha-toxin in the virulence of type A infectious diseases is limited. However, elucidation of its mechanism of action at the molecular and cellular levels is critical if novel pharmacological inhibitors or toxin molecules with modified specificity are to be designed. Some progress has been made in developing vaccines against the *C*-domain of alpha-toxin, and vaccine candidates are available to combat type A infection. Further investigation into the action of alpha-toxin will help understand the molecular mechanisms involved in infectious diseases caused by *C. perfringens* type A.
